# CD18 (ITGB2) expression in chronic lymphocytic leukaemia is regulated by DNA methylation-dependent and -independent mechanisms

**DOI:** 10.1111/bjh.13188

**Published:** 2014-10-17

**Authors:** Evelyn Hutterer, Daniela Asslaber, Chiara Caldana, Peter W Krenn, Antonella Zucchetto, Valter Gattei, Richard Greil, Tanja N Hartmann

**Affiliations:** 1 Laboratory for Immunological and Molecular Cancer Research, 3rd Medical Department with Haematology, Medical Oncology, Haemostaseology, Infectiology and Rheumatology, Oncologic Centre, Paracelsus Medical UniversitySalzburg, Austria; 2 Salzburg Cancer Research InstituteSalzburg, Austria; 3 Clinical and Experimental Onco-Haematology Unit, Centro di Riferimento OncologicoAviano, Italy

**Keywords:** chronic lymphocytic leukaemia (CLL), microenvironment, LFA-1 (CD11A/CD18;ITGAL/ITGB2), DNA methylation, proliferation

The integrin lymphocyte function-associated antigen 1 (LFA-1; CD11A/CD18; ITGAL/ITGB2), is a key regulator of lymphocyte trafficking, activation and prolonged lymphocyte residence in lymph nodes (LNs) (Reichardt *et al*, 2013); yet, little is known about the regulation of *ITGAL* (CD11A) and *ITGB2* (CD18) transcription.

Chronic lymphocytic leukaemia (CLL) cells strongly depend on the lymphoid microenvironment, where they transiently localize to receive supportive signals by accessory cells (Burger & Gribben, 2014). Previously, we found that the majority of CLL cells expressed low surface LFA-1 levels as the result of reduced *ITGB2* transcription, which was accompanied by impaired LN homing (Hartmann *et al*, 2009).

Dissecting LFA-1 expression in CLL subgroups of different cytogenetics, we observed comparable and correlating CD11A and CD18 surface expression, allowing cytometrical measurements of each subunit as a surrogate for the other (Fig1A). We found a prominent increase of CD11A and CD18 expression in Trisomy 12 (tri12) harbouring CLL (Fig1B,C), in accordance to a recent observation (Riches *et al*, 2014). The data confirmed our previous observation of associated expression of LFA-1 with CD38 and CD49D (ITGA4) (Hartmann *et al*, 2009), while we found no differences in CLL subgroups defined by the *IGHV* mutational status or ZAP70 expression (Figure S1A,B). Curiously, we observed lower CD11A levels in subgroups harbouring the unfavourable 17p deletion or the favourable 13q deletion but no association of 11q deletion with CD11A expression (Figure S1C).

**Figure 1:**
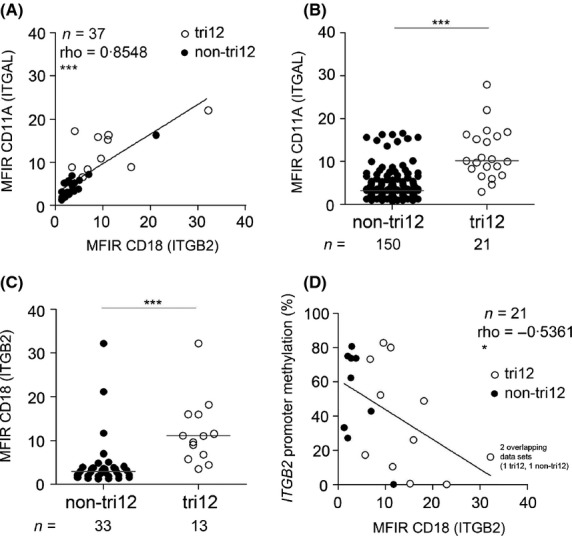
LFA-1 expression in non-tri12/tri12 CLL cells and *ITGB2* promoter methylation. LFA-1 subunit expression (CD11A/CD18) was measured by flow cytometry. CLL cells were defined by gating on viable CD19+/CD5+ cells. (A) CD11A and CD18 expression levels of CLL cells were cytometrically detected and correlated to each other. (B) CD11A expression of CLL patients with (tri12) or without tri12 (non-tri12). (C) CD18 expression of non-tri12 and tri12 CLL patients. (D) CD18 protein expression was cytometrically determined and correlated to the DNA methylation state of the *ITGB2* gene promoter. The percentage of methylation is given for the promoter region containing CpG4-CpG15 of the *ITGB2* promoter. Note that due to identical values, one non-tri12 data point (•) is overlapped by a tri12 data point (○), as indicated in the diagram. Statistics was performed using graphpad prism 5.0 (GraphPad Software, La Jolla, CA, USA). Normality was tested using D'Agostino and Pearson omnibus normality test. Mann–Whitney *t*-test was used to compare groups. Spearman Correlation was used for correlation analysis. *P*-values are depicted as: *P *≥ 0·05 ns (not significant), *P* < 0·05 *, *P* < 0·01 **, *P* < 0·001 ***. Medians are marked as lines. Corresponding numbers (*n*) of samples are given for each group in the graphs. Closed circles (•) indicate non-tri12 CLL samples, open circles (○) indicate tri12 CLL samples. MFIR, median fluorescence intensity ratio (MFI specific Antibody/MFI corresponding isotype control).

Tri12 defines a CLL subgroup with high cell proliferation and an increased frequency of Richter transformation, which manifests with lymphadenopathy and LN infiltration (Tsimberidou & Keating, 2005). Recently, we reported that tri12 CLL cells are also characterized by high CD49D expression due to *ITGA4* (CD49D) promoter hypomethylation (Zucchetto *et al*, 2013). Hypothesizing a similar mechanism responsible for regulation of LFA-1 expression, we studied promoter methylation of the rate-limiting CD18 subunit in CLL.

The *ITGB2* promoter contains 23 potentially methylated CpG motifs (CpG1-CpG23) (Agura *et al*, 1992). We analysed these sites by bisulfite conversion of genomic DNA from anti-CD19 purified CLL cells followed by nested polymerase chain reaction, cloning and sequencing. Notably, whereas the region closer to the transcription start site (CpG16-CpG23) was unmethylated in CLL, the region from •603 bp to •241 bp upstream of the transcription start site (CpG4-CpG15) was variably methylated (Figure S2). The grade of methylation in these sites inversely correlated with CD18 surface expression (Fig1D). A high grade of *ITGB2* promoter methylation of CpG4-CpG15 was found in CLL samples with low CD18 expression, whereas CD18 high expressing CLL cells, overrepresented in the tri12 CLL group even in presence of a low percentage of tri12+ cells, were mostly unmethylated at the same sites (Figs1D and S2). Our data indicate regulation of *ITGB2* transcription by DNA hypomethylation in quiescent CLL cells of this specific subgroup.

Next, we addressed whether CLL cell activation could influence CD18 expression. Therefore, CLL cells were co-cultured *in vitro* with autologous T cells on a layer of murine fibroblasts and stimulated with IL2/CpG. After a 5-d co-culture, levels of the activation marker CD86 were measured and an up-regulation of CD18 in CD86-positive CLL cells was detected (Figure S3). Particularly, in tri12 CLL, CD86-positive CLL cells in both unstimulated and IL2/CpG-stimulated samples expressed significantly higher CD18 than CD86-negative sub-clones, indicating a higher propensity of tri12 CLL to become activated and up-regulate CD18 expression, even in the absence of strong stimulation (Figure S3). Furthermore, actual cell division occurred on day 5, with significantly higher proliferation rates of tri12 CLL cells (Fig2A).

**Figure 2:**
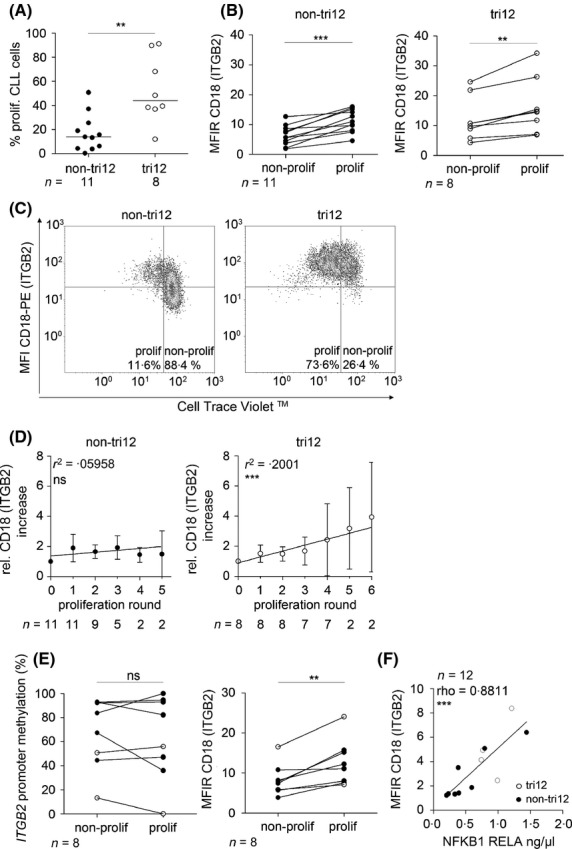
Influence of proliferation inducing stimuli on CLL cells and their LFA-1 expression. CLL cells were co-cultured with murine fibroblasts and proliferation was induced by IL2/CpG and quantified by Cell Trace Violet™ (CTV) dilution in non-tri12 and tri12 CLL samples on day 5. CLL cells were defined by gating on viable CD5+/CD19+ cells. LFA-1 expression was cytometrically determined using an anti-CD18 antibody and corresponding isotype control. (A) Proliferation of non-tri12 and tri12 CLL cells was cytometrically quantified. (B) CD18 expression of non-proliferating and proliferating CLL sub-fractions of individual samples was measured upon IL2/CpG stimulation. (C) Cytometrical density plots demonstrating CD18 expression and CTV intensity for representative non-tri12 and tri12 samples. (D) Relative CD18 expression of CLL cells undergoing subsequent proliferation rounds. *N* indicates the number of samples capable of undergoing each round of proliferation. Linear regression; circles represent means ± SD. CD18 levels were normalized to resting controls. (E) Non-proliferating and proliferating CLL subpopulations were sorted on basis of their CD5 and CD19 expression and CTV intensity using a BD Aria cell sorter (Becton, Dickinson & Company, Franklin Lakes, NJ, USA). DNA methylation and CD18 expression was analysed in the so defined CLL cells. (F) CD18 levels of CLL cells were correlated with nuclear NFKB1 subunit RELA, measured by an enzyme-linked immunosorbent assay. Statistics were performed using graphpad prism 5.0. After normality testing, Mann–Whitney *t*-test (for unpaired data; A) or Wilcoxon signed rank test (for paired data; B, E) was used to compare groups. Linear regression (D) or Spearman correlation (F) was used for correlation analysis. *P*-values are depicted as: *P *≥ 0·05 ns (not significant), *P* < 0·05 *, *P* < 0·01 **, *P* < 0·001 ***. Medians are marked as lines (A). Corresponding numbers (*n*) of samples are given for each group in the graphs. Closed circles (•) indicate non-tri12 CLL samples, open circles (○) indicate tri12 CLL samples. non-prolif, non-proliferating; prolif, proliferating; MFIR, median fluorescence intensity ratio (MFI-specific Antibody/MFI corresponding isotype control).

Within individual samples, CLL cell subpopulations that had either progressed through the cell cycle or remained quiescent showed higher CD18 expression in proliferating CLL cells (Fig2B). Remarkably, tri12 CLL were capable of undergoing more cell cycles *in vitro* than non-tri12 (Fig2C), with continuously increased CD18 levels in each proliferation round (Fig2D), suggesting a higher intrinsic proliferative capacity of CD18-high-expressing sub-clones or an up-regulation of CD18 expression by signals from the co-culture.

In addition to IL2/CpG, other co-culture systems (Asslaber *et al*, 2013) effectively induced CD18 and CD11A expression in CLL, including activated autologous or allogeneic T cells (in presence of a fibroblast layer) but not CD40LG-overexpressing fibroblasts alone (Figure S4A,B). Thus, secreted cytokines rather than direct CD40/CD40LG interactions seem responsible for this phenomenon.

Next, proliferative and quiescent CLL fractions were sorted according to their Cell Trace Violet™ (CTV) intensity using fluorescence-activated cell sorting. The levels of *ITGB2* promoter methylation, however, were similar in non-proliferating and proliferating CLL cells, despite their significant up-regulation of CD18 expression (Fig2E). Thus, while basal CD18 levels in resting CLL cells appear to be regulated by promoter methylation, CD18 up-regulation during proliferation is independent of demethylation. In line with these results, similar methylation patterns of CLL cells isolated from peripheral blood and LNs were recently observed (Cahill *et al*, 2013). Thus, rather than being a general regulatory mechanism of *ITGB2* transcription, promoter hypomethylation appears to be specific for tri12 CLL.

As the *ITGB2* promoter contains a potential NFKB1 binding site (Figure S2) (Bottinger *et al*, 1994), located in the generally unmethylated region close to the transcription start site, we analysed the correlation between active NFKB1 levels and CD18 expression in resting CLL cells. RELA levels were therefore determined in nuclear fractions. Indeed, CD18 expression directly correlated with expression of the DNA-binding NFKB1 subunit RELA (Fig2F), suggesting that NFKB1 inducing signals can influence CD18 levels in CLL. As non-tri12 CLL cells mainly display low NFKB1 levels, this pathway might be particularly important in the tri12 CLL subgroup.

In summary, we demonstrated that: (i) CD11A/CD18 is constitutively overexpressed in tri12 CLL and correlates with *ITGB2* promoter hypomethylation; (ii) an activating microenvironment up-regulates CD11A/CD18 in both non-tri12 and tri12 CLL cells via a DNA methylation-independent process; (iii) tri12 CLL hereby display a higher propensity to proliferate and up-regulate CD18 in response to activation; (iv) levels of active NFKB1 and CD18 expression correlate in CLL.

Our study highlights that LFA-1 expression in CLL is subject to multiple transcriptional regulatory mechanisms, particularly in the peculiar CLL subgroup harbouring tri12.

## Supporting Information

Fig S1 LFA-1 expression, prognostic markers and cytogenetic aberrations in CLL.

Fig S2 Schematic presentation of the *ITGB2* promoter.

Fig S3 Influence of activation inducing stimuli on CLL cells and their LFA-1 expression.

Fig S4 Increase of LFA-1 expression on CLL cells upon different proliferation inducing stimuli.

## References

[b1] Agura ED, Howard M, Collins SJ (1992). Identification and sequence analysis of the promoter for the leukocyte integrin beta-subunit (CD18): a retinoic acid-inducible gene. Blood.

[b2] Asslaber D, Grossinger EM, Girbl T, Hofbauer SW, Egle A, Weiss L, Greil R, Hartmann TN (2013). Mimicking the microenvironment in chronic lymphocytic leukaemia – where does the journey go?. British Journal of Haematology.

[b3] Bottinger EP, Shelley CS, Farokhzad OC, Arnaout MA (1994). The human beta 2 integrin CD18 promoter consists of two inverted Ets cis elements. Molecular and Cellular Biology.

[b4] Burger JA, Gribben JG (2014). The microenvironment in chronic lymphocytic leukemia (CLL) and other B cell malignancies: insight into disease biology and new targeted therapies. Seminars in Cancer Biology.

[b5] Cahill N, Bergh AC, Kanduri M, Goransson-Kultima H, Mansouri L, Isaksson A, Ryan F, Smedby KE, Juliusson G, Sundstrom C, Rosen A, Rosenquist R (2013). 450K-array analysis of chronic lymphocytic leukemia cells reveals global DNA methylation to be relatively stable over time and similar in resting and proliferative compartments. Leukemia.

[b6] Hartmann TN, Grabovsky V, Wang W, Desch P, Rubenzer G, Wollner S, Binsky I, Vallon-Eberhard A, Sapoznikov A, Burger M, Shachar I, Haran M, Honczarenko M, Greil R, Alon R (2009). Circulating B-cell chronic lymphocytic leukemia cells display impaired migration to lymph nodes and bone marrow. Cancer Research.

[b7] Reichardt P, Patzak I, Jones K, Etemire E, Gunzer M, Hogg N (2013). A role for LFA-1 in delaying T-lymphocyte egress from lymph nodes. The EMBO Journal.

[b8] Riches JC, O'Donovan CJ, Kingdon SJ, McClanahan F, Clear AJ, Neuberg DS, Werner L, Croce CM, Ramsay AG, Rassenti LZ, Kipps TJ, Gribben JG (2014). Trisomy 12 chronic lymphocytic leukemia cells exhibit upregulation of integrin signaling that is modulated by NOTCH1 mutations. Blood.

[b9] Tsimberidou AM, Keating MJ (2005). Richter syndrome: biology, incidence, and therapeutic strategies. Cancer.

[b10] Zucchetto A, Caldana C, Benedetti D, Tissino E, Rossi FM, Hutterer E, Pozzo F, Bomben R, Dal Bo M, D'Arena G, Zaja F, Pozzato G, Di Raimondo F, Hartmann TN, Rossi D, Gaidano G, Del Poeta G, Gattei V (2013). CD49d is overexpressed by trisomy 12 chronic lymphocytic leukemia cells: evidence for a methylation-dependent regulation mechanism. Blood.

