# Supplementary figures and images for: CD18 (ITGB2) expression in chronic lymphocytic leukaemia is regulated by DNA methylation-dependent and -independent mechanisms
Source: Br J Haematol. 2014 Oct 17;169(2):286–9. doi: 10.1111/bjh.13188 (PMC4406159; doi:10.1111/bjh.13188)

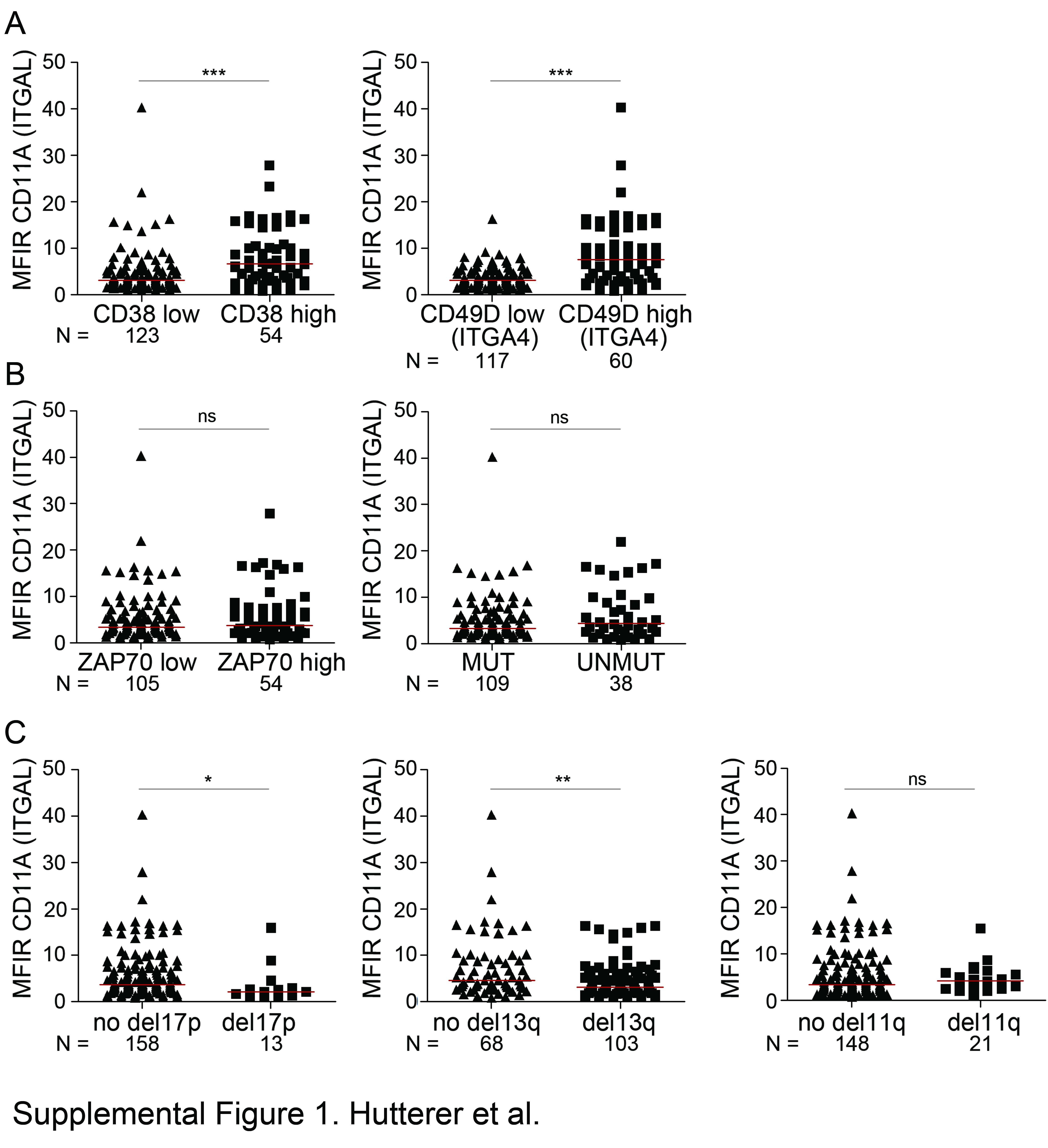

Supplement: Fig S1 — LFA-1 expression, prognostic markers and cytogenetic aberrations in CLL. [file bjh0169-0286-sd1.tiff]

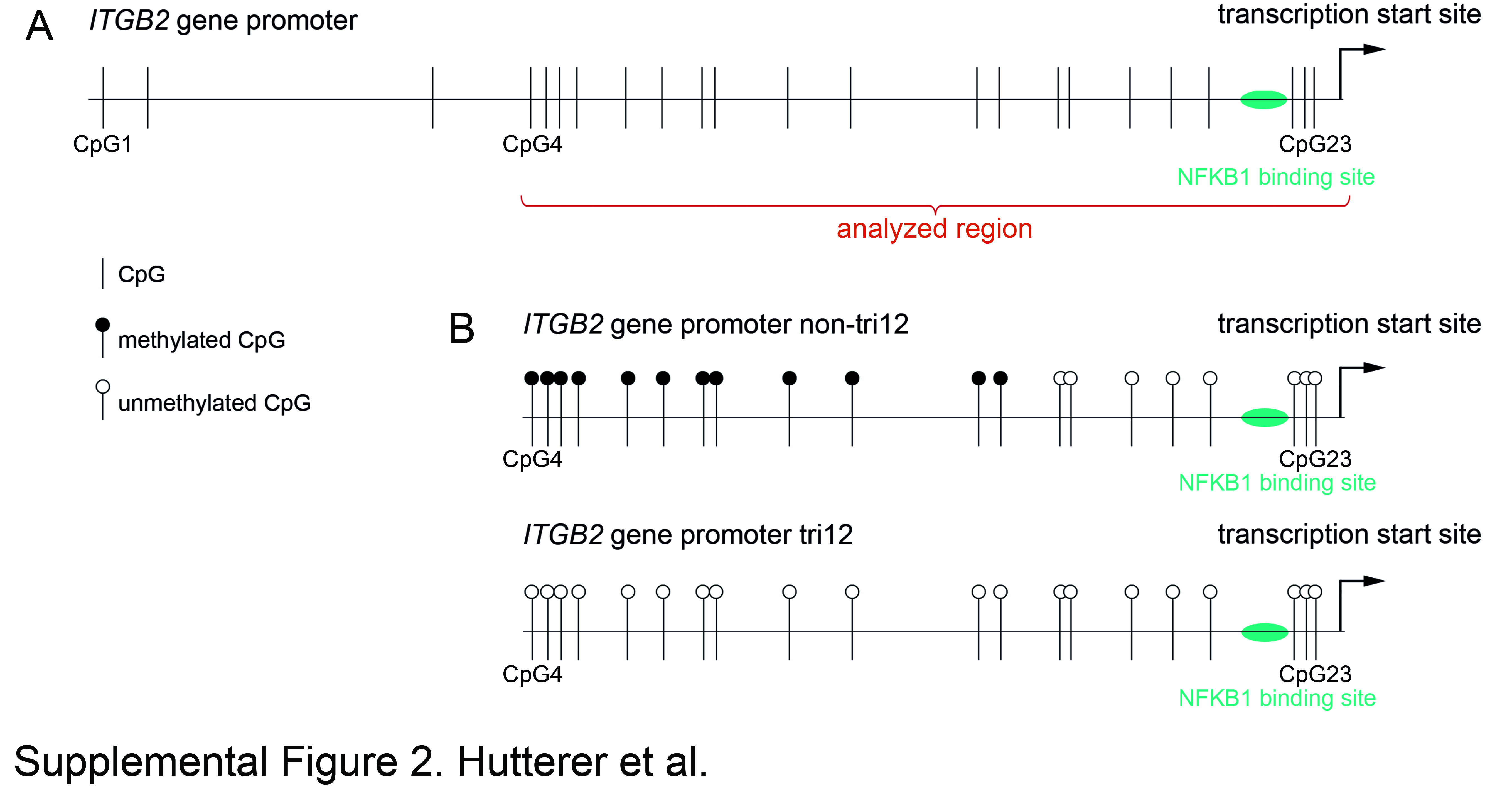

Supplement: Fig S2 — Schematic presentation of the ITGB2 promoter. [file bjh0169-0286-sd2.tiff]

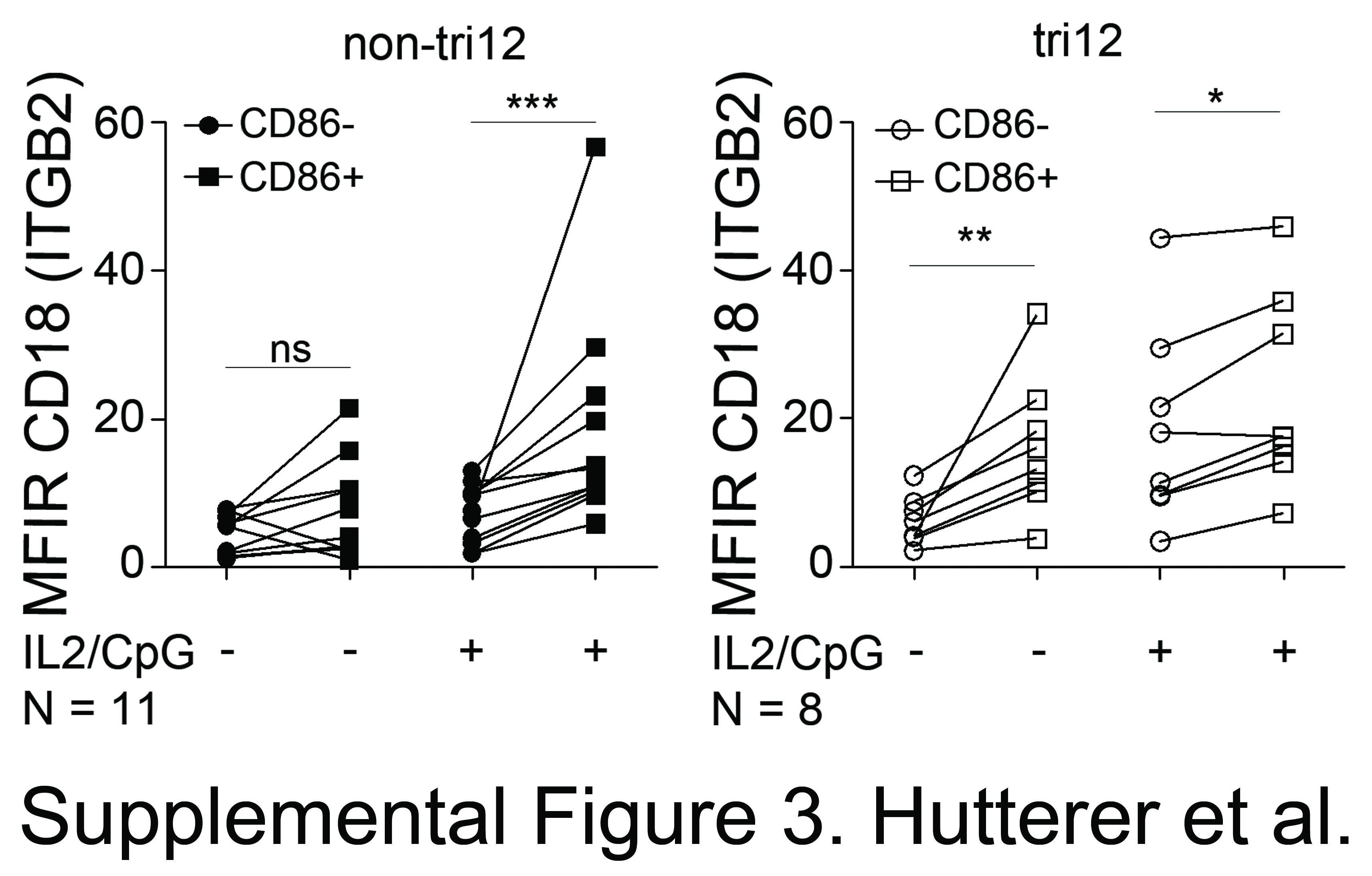

Supplement: Fig S3 — Influence of activation inducing stimuli on CLL cells and their LFA-1 expression. [file bjh0169-0286-sd3.tiff]

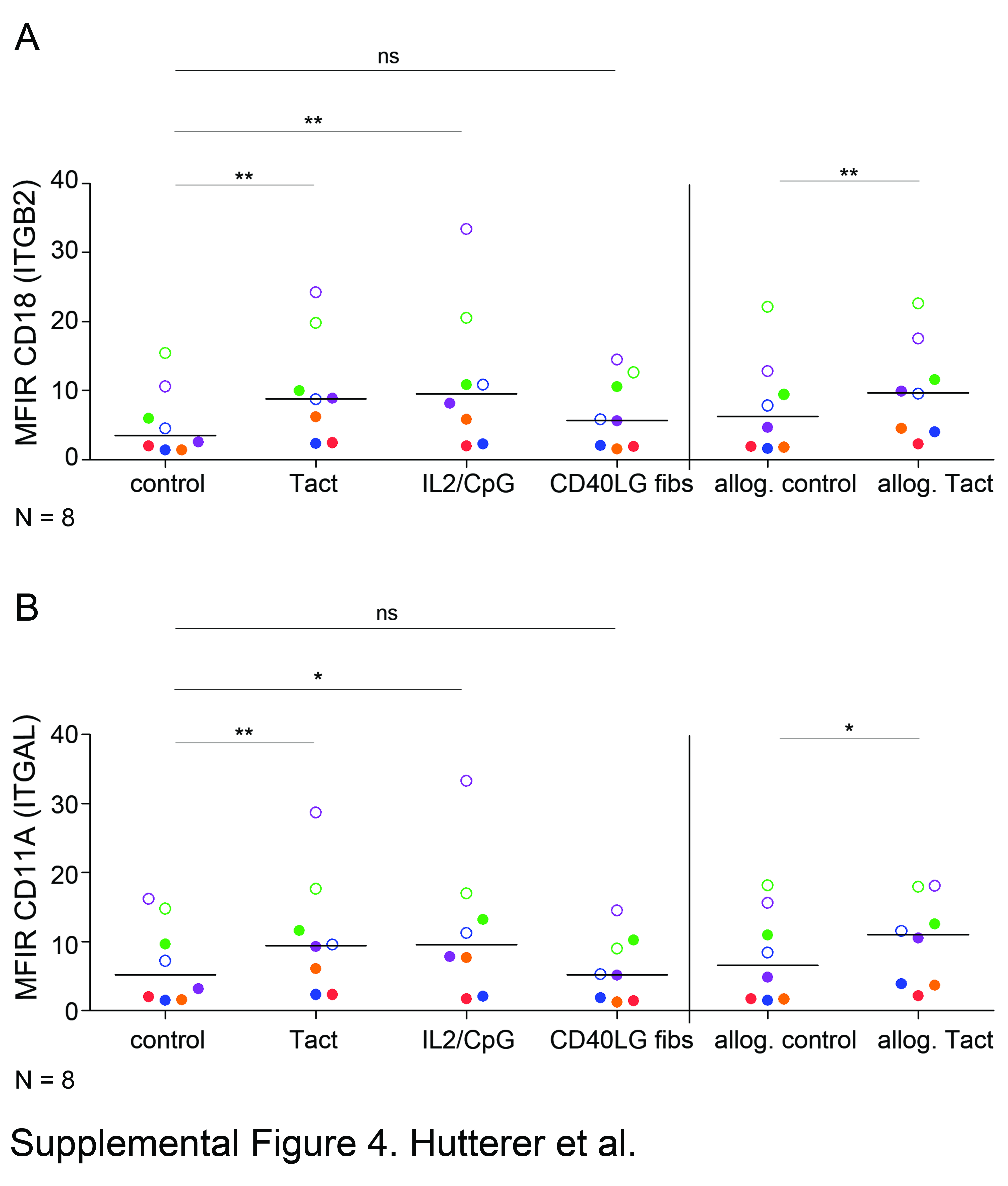

Supplement: Fig S4 — Increase of LFA-1 expression on CLL cells upon different proliferation inducing stimuli. [file bjh0169-0286-sd4.tiff]
