# Supplementary material for: CD18 (ITGB2) expression in chronic lymphocytic leukaemia is regulated by DNA methylation-dependent and -independent mechanisms
Source: Br J Haematol. 2014 Oct 17;169(2):286–9. doi: 10.1111/bjh.13188 (PMC4406159; doi:10.1111/bjh.13188)
Supplement: Supplementary file 5 [file bjh0169-0286-sd5.docx]

Supplemental Figure 1. LFA-1 expression, prognostic markers and cytogenetic aberrations in CLL. LFA-1 (CD11A; ITGAL) expression in samples of clinical low and high risk CLL groups defined by (A) CD38 and CD49D (ITGA4) (expression on < 30% versus ≥ 30% of CLL cells) (B) ZAP70 expression (high risk, NKT/B ratio < 3.1; low risk NKT/B ratio > 3.5) and the mutational status of the *IGHV* genes of the B cell receptor (mutated, sequence homology with the germline equivalent ≤ 98 %; unmutated >98 %) (C) chromosomal aberrations deletion (del)17p, del13q or del11q. Cytogenetics, *IGHV* mutations, CD49D, CD38, and ZAP70 expression were routinely evaluated as previously described (Brachtl, et al 2011). Statistics was performed using GraphPad Prism 5.0. Normality was tested using D'Agostino & Pearson omnibus normality test. Mann Whitney t-test was used to compare groups with p-values depicted as: p ≥ 0.05 ns (not significant), p < 0.05 *, p < 0.01 **, p < 0.001 ***. Medians are marked as lines. Corresponding numbers (N) of samples are given for each group in the graphs. Abbreviations: MUT = mutated; UNMUT = unmutated; MFIR = median fluorescence intensity ratio (MFI specific Antibody/MFI corresponding isotype control).

Supplemental Figure 2. Schematic presentation of the *ITGB2* promoter. (A) The scheme depicts the localization of the 23 CpGs comprising the CpG island and the NFKB1 binding site in the *ITGB2* promoter sequence according to *CpGviewer* analysis ([www.dna.leeds.ac.uk/cpgviewer/](http://www.dna.leeds.ac.uk/cpgviewer/)). (B) Representative methylation patterns of non-trisomy 12 (non-tri12) and trisomy 12 (tri12) CLL samples in the analyzed *ITGB2* promoter region from CpG4 to CpG23.

Supplemental Figure 3. Influence of activation inducing stimuli on CLL cells and their LFA-1 expression. CLL cells were co-cultured with murine fibroblasts and activated by IL2/CpG and quantified by CD86-staining in non-tri12 and tri12 CLL samples on day 5. CLL cells were defined by gating on viable CD5+/CD19+ cells. Activation and CD18 (ITGB2) expression was cytometrically determined using anti-CD86 and anti-CD18 antibodies and corresponding isotype controls. CD18 expression of resting (CD86-negative) and activated (CD86-positive) CLL sub-fractions of individual samples was measured upon IL2/CpG stimulation. Statistics was performed using GraphPad Prism 5.0. After normality testing, Wilcoxon signed rank test was used to compare groups. P-values are depicted as: p ≥ 0.05 ns (not significant), p < 0.05 *, p < 0.01 **, p < 0.001 ***. Corresponding numbers (N) of samples are given for each group in the graphs. Closed circles (●) indicate non-tri12 CLL samples, open circles (○) indicate tri12 CLL samples. Abbreviations: MFIR = median fluorescence intensity ratio (MFI specific Antibody/MFI corresponding isotype control).

Supplemental Figure 4. Increase of LFA-1 expression on CLL cells upon different proliferation inducing stimuli. (A) Peripheral blood mononuclear cells (PBMCs) from CLL patients, containing at least 5% T cells, were cultured with murine fibroblasts (control). Different activating signals were used to induce proliferation and CD18 (ITGB2) expression was measured. 1) αCD3/αCD28 beads (Dynabeads® Human T-Activator, Life Technologies) were added according to the manufacturer’s instructions to activate autologous T cells (Tact). 2) Purified CD4+ allogeneic T cells from healthy donors were added to the CLL PBMCs and fibroblasts, and αCD3/αCD28 beads were used as activating signal (allog. control; allog. Tact, respectively). 3) 100 ng/ml IL2 and 1 µM CpG (IL2/CpG), in the presence of murine fibroblasts, was used as activating signal. 4) CLL PBMCs were co-cultured with murine fibroblasts transfected with human CD40LG (CD40LG fibs). (B) CD11A (ITGAL) expression of CLL cells stimulated as described in A) was cytometrically determined. Statistics was performed using GraphPad Prism 5.0. Wilcoxon signed ranked test for paired data was used to compare stimulated samples and their corresponding controls with p-values depicted as: p ≥ 0.05 ns (not significant), p < 0.05 *, p < 0.01 **, p < 0.001 ***. Medians are marked as lines. Corresponding numbers (N) of samples are given for each group in the graphs. Closed circles (●) indicate non-trisomy 12 samples, open (○) circles indicate trisomy 12 samples. Each coloured point indicates one individual CLL sample. Abbreviations: Tact = activated T cells; CD40LG fibs = CD40LG expressing fibroblasts; allog. = allogeneic; MFIR = median fluorescence intensity ratio (MFI specific Antibody/MFI corresponding isotype control).

References:

Brachtl, G., Sahakyan, K., Denk, U., Girbl, T., Alinger, B., Hofbauer, S.W., Neureiter, D., Hofbauer, J.P., Egle, A., Greil, R. & Hartmann, T.N. (2011) Differential Bone Marrow Homing Capacity of VLA-4 and CD38 High Expressing Chronic Lymphocytic Leukemia Cells. PLoS One, 6, e23758.
